# 417. Factors associated with 30-day Mortality in Carbapenems-resistant Enterobacteriaceae Infections

**DOI:** 10.1093/ofid/ofac492.494

**Published:** 2022-12-15

**Authors:** Naphol Osaithai, Atibordee Meesing, Apichart So-Ngern

**Affiliations:** Khon Kaen university, A. Muang, Khon Kaen, Thailand; Khon Kaen university, A. Muang, Khon Kaen, Thailand; Khon Kaen university, A. Muang, Khon Kaen, Thailand

## Abstract

**Background:**

Carbapenem-resistant Enterobacteriaceae (CRE) infections are the serious conditions and provide high mortality. The studies of risk factors of death in CRE infection have been limited particularly in our center. Hence, the study was conducted for evaluating mortality and factors associated with CRE infection.

**Methods:**

A retrospective cohort study was conducted between January 1^st^, 2015, and December 31^st^, 2019 in sigle center medical university. All patients diagnosed CRE infections who aged ≥ 18 years were included and reviewed. The multivariate logistic regression was used for evaluating the factors associated with 30-day mortality.

**Results:**

Ninety-three patients enrolled in the study. The 30-day mortality occurred in thirty-five patients (37.6%). The non-survive patients had significantly higher simplified acute physiology (SAP) II score, sepsis at the time of diagnosis, and pneumonia (*p*< 0.05) than survive patients. The most common pathogen was *Klebsiella pneumoniae*, *Escherichia coli* and *Enterobacter* spp. The most common CRE isolates were susceptible to fosfomycin, tigecycline, and colistin (96.7%, 95.7%, 90.3%, respectively). The independent factors associated with 30-day mortality were having simplified acute physiology (SAP) II score > 30 [adjusted odds ratio (aOR) of 12.98 (95% confidence interval (CI), 2.84-59.34, *p*=0.001)], sepsis at the time of diagnosis [aOR of 4.77 (CI 1.19-19.20, *p*=0.03)], pneumonia [aOR of 4.64 (CI 1.31-16.47, *p*=0.01)], and improper empiric antibiotic [aOR of 7.83 (CI 1.84-33.37, *p*=0.01)].

**Figure:**
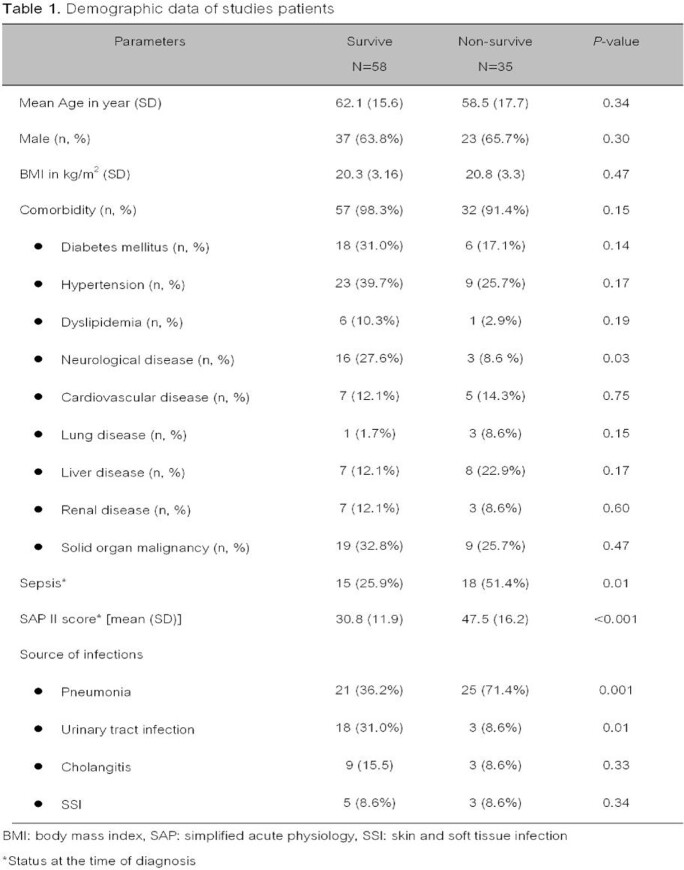

**Figure:**
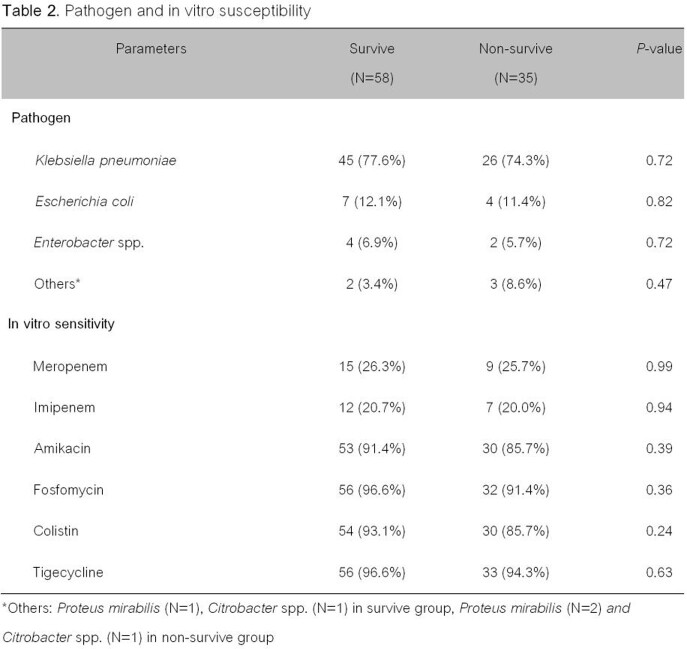

**Figure:**
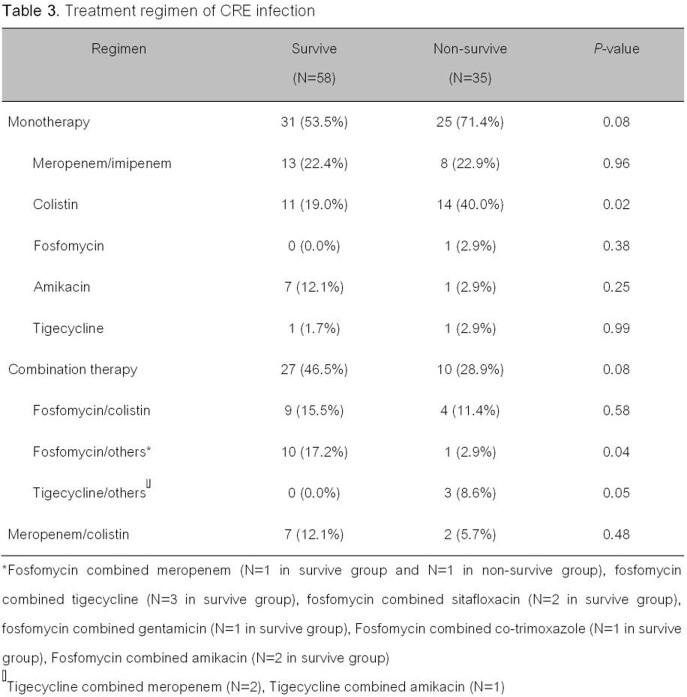

**Conclusion:**

The mortality of CRE infections were high. The factors associated with 30-day mortality were having SAP II score > 30, sepsis at the time of diagnosis, pneumonia, and improper empiric antibiotic.

**Disclosures:**

**All Authors**: No reported disclosures.

